# Profiles of FGF2, HGF, Fas/CD95, CASP9, ALDH1A1, and GLUT1 in GEP-NETs: A Comparative Tumor–Margin Study Based on Protein Concentration

**DOI:** 10.3390/ijms27135794

**Published:** 2026-06-26

**Authors:** Agata Świętek, Joanna Katarzyna Strzelczyk, Dorota Hudy, Zenon P. Czuba, Karolina Snopek-Miśta, Mariusz Kryj, Katarzyna Kuśnierz, Marcin Zeman, Władysław Skałba, Agata Abramowicz, Janusz Strzelczyk

**Affiliations:** 1 Department of Medical and Molecular Biology, Faculty of Medical Sciences in Zabrze, Medical University of Silesia in Katowice, 19 Jordana St., 41-808 Zabrze, Poland; jstrzelczyk@sum.edu.pl (J.K.S.); dorota.hudy@sum.edu.pl (D.H.); 2 Department of Microbiology and Immunology, Faculty of Medical Sciences in Zabrze, Medical University of Silesia in Katowice, 19 Jordana St., 41-808 Zabrze, Poland; zczuba@sum.edu.pl; 3 Department of Oncological Surgery, Prof. Kornel Gibiński Independent Public Central Clinical Hospital, Medical University of Silesia in Katowice, 35 Ceglana St., 40-514 Katowice, Poland; karolina.snopek@gmail.com (K.S.-M.); mariusz.kryj@sum.edu.pl (M.K.); 4 Department of Gastrointestinal Surgery, Faculty of Medical Sciences in Katowice, Medical University of Silesia in Katowice, 14 Medyków St., 40-752 Katowice, Poland; kkusnierz@sum.edu.pl; 5 III Department of Oncological Surgery, Gliwice Branch, Maria Sklodowska-Curie National Research Institute of Oncology, 15 Wybrzeże Armii Krajowej Str., 44-100 Gliwice, Poland; marcin.zeman@gliwice.nio.gov.pl (M.Z.); wladyslaw.skalba@gliwice.nio.gov.pl (W.S.); 6 Department of Clinical and Molecular Genetics, Maria Sklodowska-Curie National Research Institute of Oncology, Gliwice Branch, ul. Wybrzeże Armii Krajowej, 44-102 Gliwice, Poland; agata.abramowicz@gliwice.nio.gov.pl; 7 Department of Endocrinology and Neuroendocrine Tumors, Department of Pathophysiology and Endocrinology, Faculty of Medical Sciences in Zabrze, Medical University of Silesia in Katowice, 35 Ceglana St., 40-514 Katowice, Poland; janusz.strzelczyk@sum.edu.pl

**Keywords:** gastroenteropancreatic neuroendocrine tumors (GEP-NETs), fibroblast growth factor 2 (FGF2), hepatocyte growth factor (HGF), cluster of differentiation 95 (Fas/CD95), caspase-9 (CASP9), glucose transporter 1 (GLUT1), aldehyde dehydrogenase 1A1 (ALDH1A1), tumor, margin, protein concentration

## Abstract

Gastroenteropancreatic neuroendocrine tumors (GEP-NETs) are characterized by substantial biological heterogeneity and complex regulation of apoptosis, metabolism, and angiogenesis. The aim of this study was to evaluate the concentrations of selected proteins: FGF2, HGF, Fas/CD95, CASP9, ALDH1A1, and GLUT1 in tumor and margin samples and assess correlations with clinical and demographic parameters. A total of 59 samples from patients with GEP-NETs were analyzed using multiplex immunoassay and ELISA methods. Significant differences in protein expression between tumor and margin tissues were observed. Fas/CD95 levels were lower in tumor samples, whereas HGF concentration was higher. Elevated HGF, FGF2 and Fas/CD95 levels were associated with advanced tumor stage. HGF and GLUT1 concentrations varied depending on nodal status, while FGF2, Fas/CD95, and CASP9 levels were increased in metastatic cases. Additionally, differences related to tumor localization and the influence of smoking and alcohol consumption were identified. Dysregulation of apoptotic, metabolic, and angiogenic pathways plays a crucial role in GEP-NETs progression and highlights the importance of the tumor microenvironment. GEP-NET exhibit biological heterogeneity and complex progression driven by multiple interacting molecular pathways. The factors analyzed may have potential significance as biomarkers of disease progression; however, their exact role requires further investigation in larger, prospective cohorts.

## 1. Introduction

Gastroenteropancreatic neuroendocrine tumors (GEP-NETs) represent a distinct group of neoplasms characterized by neuroendocrine differentiation and variable clinical behavior [1]. Although many GEP-NETs demonstrate relatively slow growth, a substantial proportion exhibits invasive features, metastatic spread, or disease recurrence following surgical treatment [2]. Surgical resection with curative intent remains the primary therapeutic approach; however, prediction of biological aggressiveness and long-term outcomes based solely on histopathological criteria remains limited [3]. This has prompted increasing interest in molecular markers associated with tumor progression, metabolic adaptation, and resistance to apoptosis [4].

Growth factor signaling plays a fundamental role in tumor development and maintenance. Fibroblast growth factor 2 (FGF2) and hepatocyte growth factor (HGF) are involved in cellular proliferation, angiogenesis, and tissue remodeling, processes that are essential for tumor expansion and metastatic potential [5,6,7]. Alterations in these pathways have been reported in multiple solid tumors, suggesting their contribution to tumor–microenvironment interactions and resistance to disease progression [5,6,8,9].

Evasion of programmed cell death is another hallmark of malignancy. The cluster of differentiation 95 (Fas/CD95) receptor is a key mediator of death receptor-dependent apoptosis [10], while caspase-9 (CASP9) is a central component of the intrinsic mitochondrial apoptotic pathway [11]. Dysregulation of these proteins may impair apoptotic signaling, thereby promoting tumor cell survival and therapeutic resistance [11,12]. In neuroendocrine tumors, the status of apoptotic pathways remains incompletely characterized, particularly in the context of surrounding non-tumorous tissues [13].

Metabolic reprogramming has emerged as a critical feature of tumor biology. Glucose transporter 1 (GLUT1) facilitates increased glucose uptake and reflects enhanced glycolytic activity, which is frequently associated with tumor aggressiveness [14,15]. Aldehyde dehydrogenase 1A1 (ALDH1A1) is implicated in cellular detoxification and differentiation and has been linked to stem-like properties and an unfavorable prognosis in several cancer types [16,17]. Their expression patterns may therefore provide insight into metabolic and functional heterogeneity within GEP-NETs [2,18].

Currently, for GEP-NETs, tissue markers such as chromogranin A, synaptophysin, and especially the Ki-67 proliferation index, along with mitotic counts, form the basis of pathological diagnosis and the WHO classification, whereas circulating biomarkers have shown limited diagnostic accuracy [1,2,3,4]. Preoperative tumor grading for pancreatic neuroendocrine tumors (PanNETs) is increasingly performed using endoscopic ultrasound-guided tissue acquisition (EUS-FNA/FNB), which allows for assessment of Ki-67 grading before treatment. Good overall agreement between EUS-based grading and surgical grading has been demonstrated, although underestimation of tumor grading remains a significant limitation [19]. These findings underscore the clinical importance of reliable biomarkers and support the search for complementary markers that can improve the diagnostic and prognostic evaluation of GEP-NETs.

We hypothesized that tumor samples would demonstrate distinct expression patterns of proteins related to growth signaling, apoptosis regulation, and metabolic adaptation compared with surgical margin samples. The aim of this study was to comparatively evaluate the levels of FGF2, HGF, Fas/CD95, CASP9, ALDH1A1, and GLUT1 in tumor and matched surgical margin samples from patients with GEP-NETs. We also analyzed the association of protein concentrations with clinicopathologic variables, demographic factors, and biochemical characteristics (TNM status, G status, primary tumor location, BMI, sex, age, diabetes, hypertension, selected biomarkers, metabolic parameters, smoking status, and alcohol consumption status). Identification of such differences may contribute to a better understanding of GEP-NET pathophysiology and support the development of improved tools for disease characterization and clinical risk assessment.

## 2. Results

**Figure 1:**
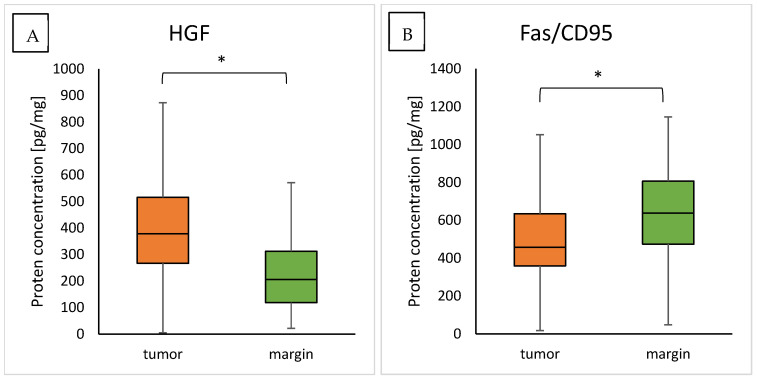
Results of the protein level analyses in tumor and margin samples. (**A**)—The Fas/CD95 level in the tumor and margin samples; (**B**)—The HGF level in the tumor and margin samples. Data were tested with the Shapiro–Wilk test for normal distribution and with the Wilcoxon signed-rank test for differences between medians. * *p*-value < 0.05.

Higher HGF concentrations were found in tumor samples than in margin samples (378.32 pg/mg (266.71–516.22) 95% IC (321.22–483.75) vs. 205.93 pg/mg (118.59–312.33) 95% IC (137.02–235.26); *p* < 0.001). The results have been presented in Figure 1A. We have found that the Fas/CD95 concentration was significantly lower in tumor samples as compared to margin samples (457.13 pg/mg (358.99–634.98) 95% IC (409.72–595.16) vs. 637.94 pg/mg (474.06–806.65) 95% IC (521.91–724.91); *p* = 0.037). The results are presented in Figure 1B.

Our analyses have shown that the FGF2 concentration in tumor samples was higher in patients with T4 status than in those with T2 and T3 (T4 vs. T2, *p* = 0.037; T4 vs. T3, *p* = 0.048). Moreover, in tumors, patients with T4 status had significantly higher levels of HGF than patients with T1 status (*p* = 0.028). The median concentration of Fas/CD95 in the tumor was significantly higher in the group with T4 compared to T1 and T2 (T4 vs. T1, *p* = 0.01; T4 vs. T2, *p* = 0.032). We also observed higher Fas/CD95 levels in the tumor samples from patients with T3 status compared to T1 and T2 status (T3 vs. T1, *p* = 0.004; T3 vs. T2, *p* = 0.013). The results are presented in Table 1.

HGF protein concentration was significantly higher in margin samples from patients with stage N1 compared to the N0 group (*p* = 0.023). However, the GLUT1 level was significantly higher in tumor samples from patients with stage N0 compared to the group with other N parameters (N0 vs. N1, *p* = 0.023; N0 vs. N2, *p* = 0.029). The results have been presented in Table 1.

Moreover, the levels of FGF2, Fas/CD95, and CASP9 were significantly higher in tumor samples obtained from patients with M1 status compared to samples from patients with M0 (FGF2, *p* = 0.010; Fas/CD95, *p* = 0.034; CASP9, *p* = 0.003). The results are presented in Table 1.

**Figure 2:**
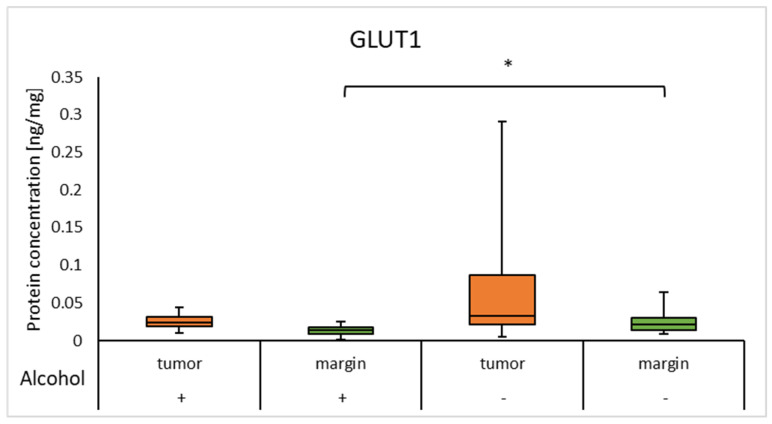
Results of the GLUT1 level analyses in tumor and margin samples compared to alcohol drinking status. Data were tested with the Shapiro–Wilk test for normal distribution and with the Mann–Whitney U test for differences between medians. * *p*-value < 0.05; + regular alcohol users; − abstinents.

In margin samples, in the group of alcohol drinkers (+), compared to non-drinkers (-), GLUT1 concentration was lower in patients who are alcohol drinkers (+), (0.01 ng/mg (0.01–0.02) 95% IC (0.001–0.025) vs. 0.02 ng/mg (0.01–0.03) 95% IC (0.017–0.027); *p* = 0.034); (Figure 2).

**Figure 3:**
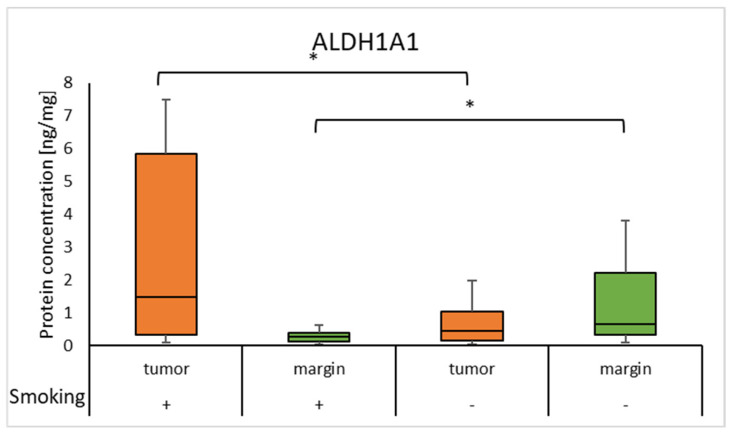
Results of the ALDH1A1 level analyses in tumor and margin samples compared to smoking status. Data were tested with the Shapiro–Wilk test for normal distribution and with the Mann–Whitney U test for differences between medians. * *p*-value < 0.05; + Smokers; − Non-smokers.

We also observed that the level of ALDH1A1 was higher in tumor samples, in the group of smokers compared to non-smokers (1.47 ng/mg (0.33–5.85) 95% IC (0.10–7.50) vs. 0.46 ng/mg (0.16–1.05) 95% IC (0.19–0.93); *p* = 0.042). On the other hand, we observed a lower concentration in of ALDH1A1 in margin samples from smokers compared to non-smokers (0.28 ng/mg (0.12–0.39) 95% IC (0.04–0.62) vs. 0.67 ng/mg (0.34–2.21) 95% IC (0.48–1.74); *p* = 0.017). The results are presented in Figure 3.

**Figure 4:**
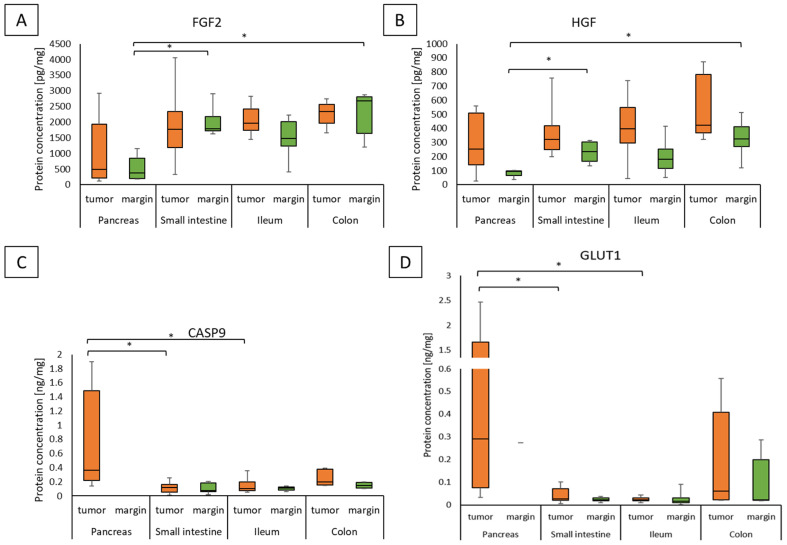
Results of the protein level analyses in tumor and margin samples compared to the localization of the primary tumor. (**A**)—The FGF2 level in the tumor and margin samples in the group of patients with tumors located in the pancreas, small intestine, ileum and colon; (**B**)—The HGF level in the tumor and margin samples in the group of patients with tumors located in the pancreas, small intestine, ileum and colon; (**C**)—The CASP9 level in the tumor and margin samples in the group of patients with tumor located in pancreas, small intestine, ileum and colon; (**D**)—The GLUT1 level in the tumor and margin samples in the group of patients with tumor located in pancreas, small intestine, ileum and colon. Data were tested with the Shapiro–Wilk test for normal distribution and with the Kruskal–Wallis test for differences between groups with Bonferroni *post hoc* to correct results for multiple comparisons. * *p*-value < 0.05.

With regard to the location of the primary tumor, in margin samples, a significantly higher concentration of FGF2 was observed in the small intestine, compared to the pancreas (1794.03 pg/mg (1718.99–2168.96) 95% IC (1629.67–2899.10) vs. 371.77 pg/mg (202.17–845.93) 95% IC (192.66–1160.01); *p* = 0.012) and between the colon and the pancreas (2678.57 pg/mg (1648.06–2811.08) 95% IC (1200.94–2866.28) vs. 371.77 pg/mg (202.17–845.93) 95% IC (192.66–1160.01); *p* = 0.008). The results are presented in Figure 4A.

We found that the concentration of HGF in margin samples was significantly higher in the small intestine, compared to samples from the pancreas (235.26 pg/mg (168.04–303.07) 95% IC (137.02–306.45) vs. 93.89 pg/mg (65.57–98.70) 95% IC (37.53–103.22); *p* = 0.040) and in colon compared to the pancreas (325.50 pg/mg (269.75–413.11) 95% IC (119.23–512.08) vs. 93.89 pg/mg (65.57–98.70) 95% IC (37.53–103.22); *p* = 0.006), as presented in Figure 4B.

A significantly higher concentration of CASP9 was observed in tumor samples in the group of patients with tumors located in the pancreas compared with the group with tumors located in the small intestine and ileum (pancreas vs. small intestine: 0.37 ng/mg (0.22–1.49) 95% IC (0.14–1.90) vs. 0.12 ng/mg (0.06–0.16) 95% IC (0.03–0.17), *p* = 0.010; pancreas vs. ileum: 0.37 ng/mg (0.22–1.49) 95% IC (0.14–1.90) vs. 0.11 ng/mg (0.07–0.20) 95% IC (0.07–0.20); *p* = 0.012). The results are presented in Figure 4C.

Similarly, we observed that the level of GLUT1 in tumor samples was higher in the patients with tumors located in the pancreas compared with the group with tumors located in the small intestine and ileum (pancreas vs. small intestine: 0.29 ng/mg (0.08–1.66) 95% IC (0.04–1.74) vs. 0.03 ng/mg (0.02–0.07) 95% IC (0.02–0.08); *p* = 0.020; pancreas vs. ileum: 0.29 ng/mg (0.08–1.66) 95% IC (0.04–1.74) vs. 0.02 ng/mg (0.02–0.03) 95% IC (0.02–0.03), *p* < 0.001), as presented in Figure 4D.

**Figure 5:**
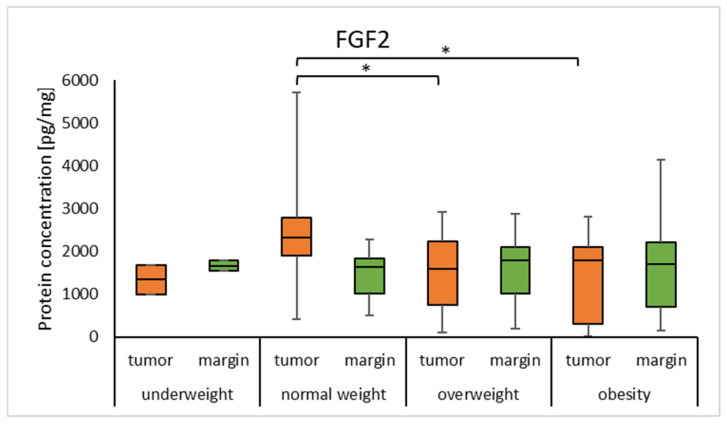
Results of the FGF2 levels in the tumor and margin samples in the groups of patients with underweight, normal weight, overweight, and obesity. Data were tested with the Shapiro–Wilk test for normal distribution and with the Kruskal–Wallis test for differences between groups with Bonferroni *post hoc* to correct results for multiple comparisons. * *p*-value < 0.05.

With regard to the BMI, in tumor samples, an increased level of FGF2 was noted in the group of patients with normal weight compared to the overweight or obesity groups (normal vs. overweight: 2317.40 pg/mg (1900.11–2782.87) 95% IC (1913.58–2625.40) vs. 1580.31 pg/mg (740.30–2242.40) 95% IC (572.97–2284.04); *p* = 0.042; normal vs. obesity: 2317.40 pg/mg (1900.11–2782.87) 95% IC (1913.58–2625.40) vs. 1801.20 pg/mg (299.19–2105.95) 95% IC (324.67–2024.27); *p* = 0.027, (Figure 5).

Moreover, the analysis of protein levels has revealed multiple significant correlations with demographic parameters, as well as between proteins, which are presented in detail in Table 2 and Table 3.

To determine whether certain parameters affected the concentrations of the studied proteins, we performed a multiple linear regression analysis. FGF2 protein levels in the tumor tissue were affected by the sex of the patients (*p* = 0.044). HGF protein levels in the tumor and margin tissues may have been dependent on the age of the patient (*p* = 0.002 and *p* = 0.035, respectively), in the margin, also on the patient’s sex, T, and G status (*p* = 0.006). M and G status had a significant effect on Fas/CD95 protein levels in the tumor (*p* < 0.001). Casp9 was affected by M status in the tumor (*p* = 0.001) and smoking status in the margin tissue (*p* = 0.028). ALDH1A1 was dependent on alcohol status in tumor tissue (*p* = 0.017), and multiple parameters affected its concentration in margin tissue (BMI, M status, hypertension, and location; *p* = 0.009). Detailed results are presented in Table 4.

We observed no other statistically significant differences between the concentrations of the analyzed proteins and clinicopathological parameters or sociodemographic parameters.

## 3. Discussion

Recently, there has been growing interest in identifying new biomarkers that could be useful for the diagnosis and prognosis of GEP-NETs. In this study, we analyzed the expression of selected proteins associated with angiogenesis, apoptosis, and metabolism in both tumor and matched margin samples.

In our study, we observed lower Fas/CD95 levels in the tumor samples compared to the margin. To our knowledge, based on medical databases, no studies have analyzed the Fas/CD95 protein in GEP-NET yet. Reports from other types of cancer indicate that loss or reduction of Fas/CD95 expression was one of the mechanisms by which tumor cells evade apoptosis and was associated with disease progression [20]. In this context, lower Fas/CD95 levels in the tumor may reflect the selection of cells resistant to proapoptotic signals. Another study demonstrated that during neoplastic transformation, Fas/CD95 expression in tumor cells gradually decreases, reducing their sensitivity to apoptosis induced by cytotoxic lymphocytes. This may reflect one of the key mechanisms of tumor escape from immune surveillance [21]. Moreover, Fas/CD95–FasL signaling may facilitate immune evasion by inducing apoptosis of tumor-infiltrating lymphocytes and limiting their accumulation within the tumor microenvironment [22,23]. Accumulating evidence suggests that retained Fas/CD95 signaling may promote tumor progression through nonapoptotic pathways, including activation of MAPK, NF-κB, and PI3K/AKT. These mechanisms have been linked to increased migration, invasion, and metastatic potential of tumor cells [10,24]. Reduced Fas/CD95 expression in the tumor versus the margin in our study may therefore reflect a balance between escaping apoptosis and preserving pro-survival signaling, although the role of non-apoptotic Fas/CD95 signaling in GEP-NETs requires further investigation. At the same time, we demonstrated higher Fas/CD95 levels in more advanced tumors, T4 vs. T1/T2, and in patients with distant metastases, M1 vs. M0, which may indicate a complex, context-dependent role for this protein. Literature data support the concept that Fas/CD95 may exert a dual role in GEP-NETs, regulating apoptosis while also contributing to tumor progression and immune escape through extra-apoptotic mechanisms [10,25].

We also demonstrated a higher concentration of HGF in the tumor compared to the margin. The literature indicates that HGF plays a key role in the processes of proliferation, cell survival, and tumor angiogenesis, acting in both autocrine and paracrine manners [26,27]. In neuroendocrine tumors, such as pituitary adenomas, widespread expression of HGF and its correlation with vessel density and proliferative index have been demonstrated [28]. The association between higher HGF levels, advanced local stage, and lymph node involvement observed in our study is consistent with the role of HGF/c-MET signaling and stromal HGF production, which contribute to tissue remodeling, angiogenesis, immune modulation, and premetastatic niche formation [26,27,29]. Studies also indicate that increased HGF expression correlates with a more advanced stage of disease and a poorer prognosis in various cancer types [30], which is consistent with our observations. Higher HGF levels in intestinal margins may reflect stronger paracrine interactions within the intestinal microenvironment than in pancreatic tissue. Overall, our findings support a role for HGF in GEP-NET progression at both the tumor and microenvironmental levels, potentially through enhanced angiogenesis, invasiveness, and tumor–stroma interactions.

Meta-analyses have shown that GLUT1, as the main glucose transporter, is overexpressed in tumors and reflects the enhanced glycolysis characteristic of the “Warburg effect” [31]. In lung neuroendocrine tumors, higher GLUT1 immunoexpression was correlated with greater proliferation and a more aggressive tumor phenotype [32]. However, our observation of higher GLUT1 levels in tumors from N0 patients than in N1/N2 patients contrasts with some previous reports. Previous studies in gastric cancer and PanNETs linked increased GLUT1 expression with lymph node metastasis, vascular invasion, proliferation, and metastatic potential [31,33,34]. The discrepancy may reflect the marked metabolic heterogeneity of GEP-NETs. Well-differentiated tumors may rely predominantly on glycolysis, whereas more aggressive tumors may switch to alternative metabolic pathways, resulting in relatively lower GLUT1 expression. Similar observations have been reported in other malignancies, where metastatic lesions with lower GLUT1 expression show reduced detectability in ^18^F-FDG PET (fluorodeoxyglucose positron emission tomography) [35]. Considering the influence of the tumor microenvironment, GLUT1 is regulated by hypoxia and HIF-1α, and its expression may be highest in oxygen-limited tumor regions. As the disease progresses and metastases develop, cancer cells may select for phenotypes better adapted to circulation and colonization of distant organs, which do not necessarily require high GLUT1 expression [36,37]. Moreover, significantly higher levels of GLUT1 were observed in pancreatic tumors compared to small intestinal tumors. GEP-NET studies demonstrated that GLUT1 was significantly more frequently elevated in tumors located in the pancreas, particularly those with a higher degree of proliferation and a more aggressive phenotype [38]. Our findings may therefore indicate greater metabolic demand and adaptation to hypoxia in pancreatic NETs. In turn, lower GLUT1 levels in margins from alcohol consumers may result from ethanol-induced impairment of glucose transport and membrane signaling, consistent with reports showing reduced glucose uptake after chronic alcohol exposure [39,40,41,42].

Our results show that FGF2 levels were significantly higher in tumors in the group of patients with a higher local stage T4 compared with those with T2 and T3 stages, as well as in patients with distant metastases compared to those without metastases. Moreover, we reported higher concentrations of FGF2 in the small intestine than in the pancreas, and higher concentrations in the colon than in the pancreas. FGF2 is a key mediator of angiogenesis, stimulating endothelial proliferation and neovascularization, and increased expression of this factor has been associated with aggressive behavior in neuroendocrine tumors [43,44,45]. The higher FGF2 levels observed in M1 patients may reflect a synergistic interaction with other angiogenic mediators, including VEGF, which facilitates angiogenesis and metastatic dissemination [46,47]. Similarly, these mechanisms can explain higher FGF2 concentrations in the intestine, which is more vascularized and has a more intense microenvironment, especially in the context of tumors with a higher degree of proliferation or angiogenesis. The richer vascular network of the intestine may also contribute to the higher FGF2 concentrations observed at this site [48]. A study on pituitary neuroendocrine tumors demonstrated that elevated FGF2 levels are associated with a more invasive tumor phenotype, supporting the relationship between FGF2 and local stage observed in our study [49]. Our study also demonstrated a statistically significant inverse correlation between BMI and FGF2 concentration in GEP-NET tumor homogenates. This finding may be related to the “obesity paradox,” whereby leaner patients may exhibit greater local tumor aggressiveness and proangiogenic activity [50]. Alternatively, adipose tissue-derived FGF2 in overweight individuals may reduce the need for local tumor production, whereas lean patients may rely more strongly on autocrine FGF/FGFR activation to sustain angiogenesis [43,51].

In this study, we also observed statistically higher CASP9 levels in primary tumors from patients with distant metastases compared to those without metastases. Based on medical databases, we found no studies analyzing the CASP9 protein in GEP-NET. A growing body of research indicates that the role of CASP9 in cancer is more complex and is not limited solely to the induction of apoptosis. Emerging evidence indicates that CASP9 has functions beyond apoptosis, including regulation of endosomal trafficking and lysosomal activity, which may support tumor cell adaptation [52]. The effects of CASP9 on tumor progression appear to be context-dependent, with both pro- and anti-tumor activities reported [53]. Moreover, in the tumor environment, where numerous interactions with the immune system and microenvironmental factors occur, CASP9 signaling may modulate immune responses and facilitate tumor immune escape [54]. The increased CASP9 expression observed in metastatic GEP-NETs may reflect enhanced cellular stress and activation of apoptotic pathways, while resistance to downstream apoptotic mechanisms could permit tumor cell survival despite increased expression of apoptosis initiators [55]. Moreover, the higher concentration of CASP9 in pancreatic tumors compared to that in intestinal tumors, as demonstrated in our study, may indicate site-specific differences in mitochondrial apoptotic signaling and cellular stress responses [34]. ALDH1A1 is a key enzyme responsible for the detoxification of aldehydes, the main components of tobacco smoke [16,17,56]. Higher levels of this protein in tumors of smokers may represent a defensive response of the tumor to chemical stress, aimed at neutralizing the toxic products of tobacco combustion. Smoking has also been associated with enhanced stem cell-like properties and tumor aggressiveness [57]. In addition, ALDH1A1 is widely recognized as a marker of cancer stem cell-like populations and has been associated with enhanced self-renewal capacity, therapeutic resistance, and metastatic potential in several malignancies. Therefore, the increased ALDH1A1 expression observed in GEP-NETs, particularly in smokers, may reflect enrichment of a stem cell-like subpopulation contributing to a more aggressive tumor phenotype. Although the precise role of ALDH1A1-positive cells in GEP-NET biology remains to be elucidated, our findings support their potential biological and clinical relevance [58]. Conversely, reduced ALDH1A1 levels in the margins of smokers may reflect field cancerization and impaired local defense mechanisms caused by chronic exposure to tobacco carcinogens, potentially representing an early marker of genomic instability [59,60,61,62].

The analysis revealed significant correlations between protein levels and selected demographic and biochemical parameters, including age, BMI, and serotonin concentration. The positive correlation of FGF2, Fas/CD95, and HGF with age may reflect age-related remodeling of the tumor microenvironment. This observation is consistent with the senescence-associated secretory phenotype (SASP), characterized by increased production of growth factors and mediators involved in angiogenesis, stromal remodeling, and immune regulation [17,63,64]. Another significant observation in our study was the positive correlation of ALDH1A1 concentration in the tumor with BMI. In addition to its role in detoxification, ALDH1A1 also participates in retinoid metabolism and adipocyte differentiation [65,66]. Literature data suggest that this association may reflect the involvement of this enzyme in both detoxification and metabolic regulation, supporting a link between host metabolic status and GEP-NET biology [56,65,66]. In turn, the strong negative correlation between GLUT1 in the margin and blood serotonin levels reflects the influence of biogenic amines on glucose metabolism in the tumor microenvironment. Serotonin, frequently overproduced by neuroendocrine tumors, regulates insulin sensitivity, glucose transport, and GLUT expression, potentially contributing to the reduced GLUT1 levels observed in the margin tissue of patients with high serotonin secretion [67,68,69,70,71].

To sum up, he observed correlations between protein expression patterns support the concept that angiogenic, metabolic, and apoptotic pathways are closely interconnected and coordinately activated in GEP-NETs. Crosstalk between the FGF/FGFR, HGF/MET, Fas/CD95, CASP9, GLUT1, and ALDH1A1 pathways may collectively contribute to tumor growth, survival, and progression, reflecting the complex biology of these tumors [10,11,12,43,57,72,73,74,75].

## 4. Materials and Methods

### 4.1. Study Population

**Figure 6:**
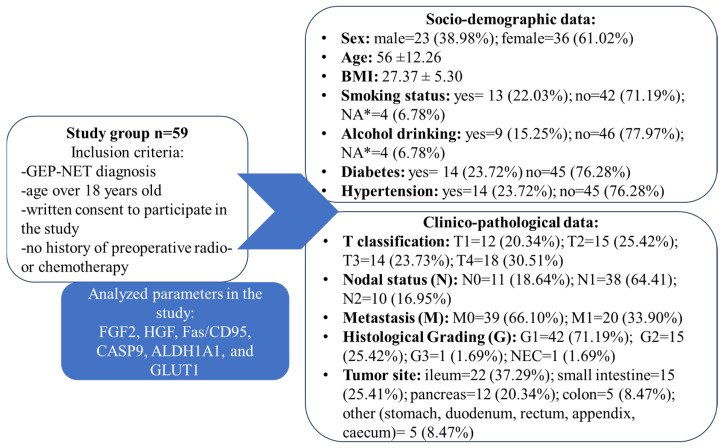
Flow chart of the study. NA * not assessed.

The study was conducted according to the guidelines of the Declaration of Helsinki and was approved by the Institutional Review Board on Medical Ethics, No. BNW/NWN/0052/KB1/126/I/22/23. The material was collected from patients treated surgically at three clinical centers: the Department of Oncological Surgery, Prof. Kornel Gibiński Independent Public Central Clinical Hospital, Medical University of Silesia in Katowice, Poland; the Department of Gastrointestinal Surgery, Faculty of Medical Sciences in Katowice, Medical University of Silesia in Katowice, Poland and the III Department of Oncological Surgery, Maria Sklodowska-Curie National Research Institute of Oncology in Gliwice, Poland. A total of 102 tissue specimens were included in the analysis, comprising 59 samples derived from GEP-NETs and 43 samples obtained from tumor-free surgical margins. In several cases, only tumor tissue was available, and corresponding margin material could not be collected. All tumor specimens underwent routine histopathological evaluation to confirm the diagnosis of GEP-NET. Surgical margin samples were independently examined by a certified pathologist to verify the absence of malignant infiltration. Tumor grading and staging were performed in accordance with the guidelines of the 9th edition of the American Joint Committee on Cancer (AJCC) Staging System for Gastroenteropancreatic Neuroendocrine Neoplasms [76]. In addition, information on the concentrations of selected biomarkers (chromogranin A (CgA), serotonin, 5-hydroxyindoleacetic acid (5-HIAA)), and metabolic parameters (glucose, cholesterol (TCH), triglycerides (TG) in serum) was extracted from hospital medical records. Ethical approval for the study was granted by the institutional Research Ethics Committee (approval number BNW/NWN/0052/KB1/126/I/22/23). Following surgical resection, all specimens were transported under appropriate conditions to the Laboratory of Medical and Molecular Biology at the Medical University of Silesia in Katowice, Poland, where they were stored at −80 °C until further molecular analyses. Detailed socio-demographic, clinico–pathological characteristics, and inclusion criteria of the study population are summarized in Figure 6 and Table S1 in the Supplementary Materials.

### 4.2. Homogenization and Total Protein Concentration

A total of 102 tumor and margin samples were homogenized according to a previously established protocol [77]. Samples were homogenized in PBS buffer using a PRO 200 homogenizer (PRO Scientific Inc., Oxford, CT, USA) at 10,000 rpm and further disrupted with an ultrasonic processor (UP100, Hilscher, Hattersheim am Main, Germany).

Total protein content was quantified by UV/VIS spectrophotometry using an ND-1000 NanoDrop system (NanoDrop Technologies, Wilmington, DE, USA). Method accuracy was verified in a randomly selected subset of 15 samples using a fluorescence-based assay (Accu Orange Protein Quantitation Kit, Biotium, Fremont, CA, USA). Fluorescence was measured with a SYNERGY H1 microplate reader (BioTek, Winooski, VT, USA) (λ_ex 480 nm, λ_em 598 nm), and data were analyzed using Gen5 2.06 software. Statistical analysis showed no significant differences between spectrophotometric and fluorometric protein measurements.

### 4.3. Multiplex Immunoassay for FGF2, HGF, Fas/CD95, and ELISA Kits for CASP9, ALDH1A1, GLUT1 Concentration

The FGF2, HGF, and Fas/CD95 concentrations were measured using the Bio-Plex 3D Suspension Array System (Bio-Rad Laboratories Inc., Hercules, CA, USA) and analyzed with Bio-Plex Manager Software version 6.0. Just before the study, the supernatants were thawed, centrifuged at 14,000 rpm for 5 min, and approximately 50 μL of each supernatant obtained was transferred to dedicated 96-well plates (LXSAHM-09, BioPlex Human Luminex Discovery Assay, Bio-Rad Laboratories Inc., Hercules, CA, USA). Selected protein concentrations were normalized to total protein content and reported as pg/mg of total protein.

CASP9, ALDH1A1, and GLUT1 levels were measured with commercial ELISA kits (SEA627Hu, SEE824Hu, and SEB185Hu; Cloud Clone, Wuhan, China) according to the manufacturer’s instructions. The assay detection limits were <0.113 ng/mL for CASP9, <0.61 ng/mL for ALDH1A1, and <0.121 ng/mL for GLUT1. Selected protein concentrations were normalized to total protein content and reported as ng/mg of total protein.

### 4.4. Statistical Analysis

The normality of variable distribution was assessed with the use of the Shapiro–Wilk test. The choice of subsequent statistical procedures was based on the obtained distribution characteristics. Differences between tumor and margin samples were tested with the Wilcoxon signed-rank test. Comparisons between two independent groups were performed using either the Mann–Whitney U test for non-normally distributed data or the Student’s t-test for normally distributed variables, enabling evaluation of differences in medians or means, respectively. In cases where more than two groups were analyzed, the Kruskal–Wallis test was applied, and when appropriate, pairwise comparisons were conducted using the Bonferroni *post hoc* procedure.

Relationships between quantitative parameters were explored by calculating Spearman’s rank correlation coefficients. Multiple linear regression was performed to determine whether more than one parameter could affect the results. Sex, age, BMI, alcohol intake, smoking status, diabetes, hypertension, TNM status, G status, and location were assessed simultaneously. The association between categorical demographic variables and tumor localization was examined using Fisher’s exact test. A *p*-value below 0.05 was considered to indicate statistical significance. Presented *p*-values in text are from the Mann–Whitney U test, Student t-test, or *post hoc* Bonferroni. Quantitative data are presented either as medians with interquartile ranges (M (Q1–Q3), 95% IC) or as means with standard deviations (M ± SD, 95% IC). Statistically significant findings are indicated graphically by asterisks in the corresponding figures.

## 5. Conclusions

The study revealed significant differences in the concentration of the analyzed proteins between tumor tissues and the margins, highlighting the importance of both cancer cells and their microenvironment in the pathogenesis of neuroendocrine tumors. Variations in protein levels depending on clinical stage (TNM) indicate that processes related to apoptosis, angiogenesis, and metabolism undergo dynamic changes as the disease progresses. Furthermore, the observed differences depending on tumor location suggest the existence of distinct biological mechanisms between tumors of different organ origins. Additionally, the influence of environmental factors, such as smoking and alcohol consumption, may modulate the expression of the studied proteins, reflecting their role in the response to metabolic and toxic stress.

The results obtained highlight the biological heterogeneity of GEP-NET and the complex nature of their progression, which stems from the interaction of multiple molecular pathways. The factors analyzed may have potential significance as biomarkers of disease progression; however, their exact role requires further investigation in larger, prospective cohorts.

## Figures and Tables

**Table 1:** Results of the protein level analyses in tumor and margin samples compared to T, N and M status.

Parameter		Concentration in Samples [Median (Interquartile Range)]								
		T1	T2	T3	T4	N0	N1	N2	M0	M1
FGF2 [pg/mg]	Tumor	1997.58 (1242.28–2625.40) 95% IC (993.52–2919.95)	1694.77 (305.58–2272.81) 95% IC (305.58)	1727.38 (572.97–2117.50) 95% IC (572.97–2117.50)	2498.86 (1829.17–5700.44) 95% IC (1536.79–5700.44)	993.52 (272.01–2174.58) 95% IC (260.82–2187.63)	1941.99 (1519.96–2572.32) 95% IC (1663.99–2317.40)	2142.44 (1681.52–2593.55) 95% IC (1536.79–2735.75)	1707.59 (783.24–2070.89) 95% IC (1261.21–2011.64)	2316.81 (1642.75–2593.55) 95% IC (1642.75–2593.55)
	Margin	1285.08 (444.61–1790.53) 95% IC (211.68–1794.03)	1797.10 (1290.43–2697.63) 95% IC (1250.53–2899.10)	1634.87 (1457.80–2099.30) 95% IC (1200.94–2226.42)	1850.42 (763.13–2476.01) 95% IC (588.18–2678.57)	1787.02 (277.46–3089.40) 95% IC (141.56–4145.43)	1634.87 (1190.70–2109.03) 95% IC (1229.61–2056.93)	1746.70 (1396.98–1962.38) 95% IC (1250.53–2093.21)	1481.93 (807.80–2039.20) 95% IC (1200.94–1831.55)	1811.95 (1345.32–2219.32) 95% IC (1250.53–2273.45)
HGF [pg/mg]	Tumor	250.72 (219.09–455.84) 95% IC (215.66–483.75)	321.22 (217.90–450.03) 95% IC (214.16–467.42)	356.35 (284.96–571.55) 95% IC (284.72–683.03)	527.04 (389.02–717.14) 95% IC (383.41–737.95)	262.98 (159.10–396.30) 95% IC (135.76–397.86)	383.41 (271.24–564.59) 95% IC (285.19–527.04)	421.30 (320.63–550.52) 95% IC (320.03–613.53)	342.65 (229.24–531.55) 95% IC (251.33–397.86)	439.48 (302.97–540.39) 95% IC (321.22–527.04)
	Margin	173.10 (109.03–307.84) 95% IC (94.17–325.52)	129.15 (103.90–247.37) 95% IC (103.22–273.35)	211.40 (137.02–306.45) 95% IC (93.60–319.93)	233.28 (211.84–334.98) 95% IC (202.59–344.45)	116.19 (37.53–135.34) 95% IC (21.34–169.45)	221.09 (158.62–316.56) 95% IC (180.83–301.94)	178.38 (102.69–353.37) 95% IC (53.10–380.13)	178.38 (107.96–315.43) 95% IC (118.38–273.35)	230.81 (133.09–319.89) 95% IC (137.02–314.28)
Fas/CD95 [pg/mg]	Tumor	347.43 (249.22–415.06) 95% IC (193.12–465.08)	397.83 (274.21–449.18) 95% IC (274.21–449.18)	642.37 (557.92–704.15) 95% IC (557.10–704.51)	545.73 (423.48–764.17) 95% IC (424.03–749.53)	368.19 (197.41–500.24) 95% IC (193.12–703.79)	469.12 (353.97–620.09) 95% IC (389.39–558.73)	576.84 (424.03–816.87) 95% IC (409.72–992.18)	420.61 (327.48–611.38) 95% IC (347.43–482.64)	551.42 (424.03–723.07) 95% IC (424.03–723.07)
	Margin	698.65 (473.30–998.27) 95% IC (413.02–1119.45)	648.85 (576.57–776.35) 95% IC (521.91–834.91)	648.92 (429.61–725.28) 95% IC (411.30–725.41)	619.73 (388.72–878.50) 95% IC (332.35–956.30)	521.91 (100.78–699.56) 95% IC (47.76–910.82)	648.85 (474.06–920.50) 95% IC (484.54–722.52)	724.91 (512.16–809.26) 95% IC (337.41–834.91)	622.02 (421.04–748.98) 95% IC (473.73–722.52)	673.78 (503.22–821.77) 95% IC (521.91–808.63)
CASP9 [ng/mg]	Tumor	0.12 (0.10–0.23) 95% IC (0.10–0.52)	0.12 (0.10–0.22) 95% IC (0.08–0.27)	0.10 (0.07–0.16) 95% IC (0.06–0.17)	0.19 (0.10–0.37) 95% IC (0.09–0.37)	0.17 (0.12–1.41) 95% IC (0.08–1.90)	0.12 (0.08–0.19) 95% IC (0.10–0.16)	0.20 (0.14–0.34) 95% IC (0.06–0.52)	0.10 (0.07–0.14) 95% IC (0.08–0.12)	0.20 (0.13–0.36) 95% IC (0.14–0.35)
	Margin	0.072 (0.044–0.08) 95% IC (0.02–0.09)	0.12 (0.11–0.17) 95% IC (0.11–0.20)	0.11 (0.08–0.13) 95% IC (0.06–0.17)	0.13 (0.11–0.20) 95% IC (0.11–0.21)	0.12 (0.07–0.15) 95% IC (0.02–0.17)	0.11 (0.09–0.19) 95% IC (0.09–0.19)	0.11 (0.07–0.14) 95% IC (0.06–0.20)	0.11 (0.07–0.13) 95% IC (0.08–0.12)	0.12 (0.11–0.19) 95% IC (0.11–0.20)
ALDH1A1 [ng/mg]	Tumor	0.34 (0.07–1.10) 95% IC (0.05–1.32)	0.47 (0.24–1.15) 95% IC (0.07–1.47)	0.36 (0.17–0.89) 95% IC (0.16–1.07)	0.93 (0.11–1.44) 95% IC (0.08–1.49)	0.29 (0.15–0.62) 95% IC (0.07–1.07)	0.44 (0.15–1.30) 95% IC (0.20–1.10)	0.71 (0.14–1.07) 95% IC (0.07–1.09)	0.41 (0.09–1.07) 95% IC (0.18–0.96)	0.66 (0.24–1.37) 95% IC (0.29–1.28)
	Margin	0.31 (0.16–2.13) 95% IC (0.04–3.82)	1.37 (0.40–2.34) 95% IC (0.28–2.44)	1.29 (0.59–3.33) 95% IC (0.12–3.65)	0.48 (0.27–0.69) 95% IC (0.26–0.71)	2.07 (0.75–3.01) 95% IC (0.31–3.33)	0.62 (0.31–2.19) 95% IC (0.31–1.74)	0.46 (0.27–1.40) 95% IC (0.26–1.50)	0.71 (0.38–2.16) 95% IC (0.46–1.74)	0.31 (0.23–0.81) 95% IC (0.18–1.37)
GLUT1 [ng/mg]	Tumor	0.03 (0.02–0.09) 95% IC (0.01–0.13)	0.22 (0.02–1.43) 95% IC (0.02–1.63)	0.03 (0.02–0.07) 95% IC (0.02–0.09)	0.03 (0.02–0.05) 95% IC (0.02–0.05)	0.22 (0.03–1.48) 95% IC (0.03–1.74)	0.03 (0.20–0.06) 95% IC (0.02–0.05)	0.02 (0.02–0.04) 95% IC (0.01–0.04)	0.03 (0.02–0.06) 95% IC (0.02–0.04)	0.04 (0.02–0.13) 95% IC (0.02–0.10)
	Margin	0.02 (0.01–0.02) 95% IC (0.001–0.03)	0.02 (0.01–0.03) 95% IC (0.01–0.04)	0.02 (0.02–0.15) 95% IC (0.01–0.17)	0.02 (0.01–0.03) 95% IC (0.01–0.03)	0.02 (0.02–0.03) 95% IC (0.02–0.04)	0.02 (0.01–0.02) 95% IC (0.01–0.02)	0.01 (0.01–0.02) 95% IC (0.01–0.04)	0.02 (0.01–0.06) 95% IC (0.01–0.04)	0.02 (0.01–0.02) 95% IC (0.01–0.03)

**Table 2:** Correlations between analyzed proteins and demographic parameters.

Protein	Variable	Sample	*p*-Value	Spearman’s rs
FGF2	Age	Tumor	0.060	0.383
Fas/CD95	Age	Tumor	0.020	0.417
HGF	Age	Margin	0.032	0.327
ALDH1A1	BMI	Tumor	0.044	0.324
GLUT1	Serotonin	Margin	0.037	−0.540

**Table 3:** Inter-protein correlations.

Protein	Protein	Sample	*p*-Value	Spearman’s rs
FGF2	HGF	Tumor/Tumor	0.047	0.297
FGF2	Fas/CD95	Tumor/Tumor	0.008	0.381
FGF2	Fas/CD95	Tumor/Margin	0.042	0.336
FGF2	CASP9	Tumor/Tumor	0.027	0.369
FGF2	CASP9	Tumor/Margin	0.013	0.469
HGF	Fas/CD95	Tumor/Tumor	0.008	0.555
HGF	Fas/CD95	Margin/Tumor	0.002	0.466
GLUT1	CASP9	Tumor/Tumor	0.029	0.380
GLUT1	ALDH1A1	Tumor/Margin	0.038	0.375
FGF2	HGF	Margin/Margin	<0.001	0.517
FGF2	Fas/CD95	Margin/Margin	0.009	0.413
FGF2	CASP9	Margin/Margin	0.001	0.597
HGF	Fas/CD95	Margin/Margin	0.060	0.416
HGF	CASP9	Margin/Margin	0.003	0.533

**Table 4:** Results of multiple linear regression analysis.

Analyzed Cytokines	Tissue	Best Fitted Predictors	R^2^	R^2^ Adjusted	b_0_	F	*p*
FGF2	tumor	sex (b = −1200.51; *p* = 0.044)	0.11	0.09	2971.12; *p* < 0.001	4.39	0.044
	margin	BMI (b = 47.26; *p* = 0.207)	0.05	0.02	521.79; *p* = 0.622	1.66	0.207
HGF	tumor	age (b = 30.05; *p* = 0.002)	0.24	0.22	−1028.44; *p* = 0.053	11.15	0.002
	margin	age (b = 3.72; *p* = 0.035); sex (b = 110.13; *p* = 0.008); T status (b = 43.63; *p* = 0.017); G status (b = −68.12; *p* = 0.045)	0.36	0.28	−92.59; *p* = 0.306	4.47	0.006
Fas/CD95	tumor	M status (b = 299.58; *p* < 0.001); G status (b = 223.09; *p* < 0.001)	0.45	0.42	118.77; *p* = 0.213	14.16	<0.001
	margin	N status (b = 102.37; *p* = 0.220)	0.04	0.02	522.62; *p* < 0.001	1.56	0.22
CASP9	tumor	M status (b = 0.13; *p* = 0.001)	0.38	0.35	0.1; *p* < 0.001	13.21	0.001
	margin	smoking status (b = 0.05; *p* = 0.028)	0.2	0.17	0.11; *p* < 0.001	5.55	0.028
ALDH1A1	tumor	alcohol status (b = 1.67; *p =* 0.017)	0.21	0.18	0.62; *p* = 0.041	6.55	0.017
	margin	BMI (b = 0.14; *p* = 0.034); M status (b = −2.68; *p* = 0.004); hypertension status (b = 1.77; *p* = 0.038); location * (b = −0.63; *p* = 0.019)	0.46	0.36	−0.38; *p* = 0.846	4.47	0.009
GLUT1	tumor	N status (b = −0.15; *p* = 0.056)	0.12	0.09	0.27; *p* = 0.005	3.96	0.056
	margin	location * (b = −0.01; *p* = 0.235)	0.05	0.02	0.06; *p* = 0.002	1.47	0.235

* Location was presented as 0-small intestine; 1-pancrease; 2-colon; 3-ileum; 4-other.

## Data Availability

The raw data supporting the conclusions of this article will be made available by the authors on request.

## Supplementary Materials

The following supporting information can be downloaded at: https://www.mdpi.com/article/10.3390/ijms27135794/s1.
