# Supplementary material for: Profiles of FGF2, HGF, Fas/CD95, CASP9, ALDH1A1, and GLUT1 in GEP-NETs: A Comparative Tumor–Margin Study Based on Protein Concentration
Source: Int J Mol Sci. 2026 Jun 26;27(13):5794. doi: 10.3390/ijms27135794 (PMC13362495; doi:10.3390/ijms27135794)
Supplement: Supplementary file 1 [file ijms-27-05794-s001.zip › ijms-4307182-supplementary.pdf]

Table S1 Description of study groups

		T				N			M		G			
		1	2	3	4	0	1	2	0	1	1	2	3	4
Patients		12	15	14	18	11	38	10	39	20	42	15	1	1
Sex	male	4	5	5	9	3	16	4	15	8	17	6	0	0
	female	8	10	9	9	8	22	6	24	12	25	9	1	1
Age		53.91+/-8.42	54.80+/-13.79	59.86+/-12.02	59.89+/-11.54	48.91+/-14.77	55.87+/-9.83	72.22+/-5.21	54.51+/-12.59	61.50+/-11.01	54.27+/-10.15	69.38+/-6.73	57	72
BMI		26.89+/-5.68	28.43+/-4.92	28.68+/-5.85	26.66+/-5.79	27.24+/-6.47	27.71+/-4.40	26.53+/-7.17	27.53+/-5.37	27.87+/-5.85	28.08+/-5.25	26.75+/-6.45	27.14	23.40
Smoking	yes	4	5	3	1	4	8	1	9	4	10	3	0	0
	no	8	9	9	16	7	29	6	27	15	30	10	1	1
Alcohol	yes	3	2	2	2	1	6	2	6	3	7	2	0	0
	no	9	12	10	15	10	31	5	30	16	33	11	1	1

		Smoking		Alcohol		Location				BMI			
		Yes	No	Yes	No	Pancrease	Small Intestine	Ileum	Colon	Underweight	Normal weight	Overweight	Obesity
Patients		13	42	9	46	12	15	22	5	2	19	16	19
Sex	male	5	18	6	17	3	6	11	1	1	7	6	8
	female	8	24	3	29	9	9	11	4	1	12	10	11
Age		59.42+/-8.40	56.02+/-12.03	56.75+/-8.41	56.78+/-11.85	50.00+/-12.12	57.53+/-13.86	58.05+/-11.09	57.40+/-14.62	47.00+/-31.11	58.26+/-11.19	52.81+/-12.19	59.58+/-11.28
BMI		26.96+/-5.45	27.59+/-5.53	25.03+/-4.58	27.91+/-5.55	27.17+/-4.05	28.25+/-6.11	26.68+/-5.40	30.92+/-8.54	16.78+/-0.82	22.85+/-1.36	27.19+/-1.30	33.96+/-2.98
Smoking	yes	13	0	4	9	3	7	5	1	1	3	5	4
	no	0	42	5	37	9	2	16	2	1	16	11	13
Alcohol	yes	4	5	9	0	0	1	6	1	1	4	2	2
	no	9	37	0	46	12	4	15	2	1	15	14	15

Number of patients in every group or mean with standard deviation of age and BMI (body mass index (kg/m²)) for patients in group

T - primary tumor extent

N - regional lymph node involvement

M - distant metastases

G - histological grade based on mitotic count and Ki-67 proliferation index
